# Embryo Banking with Mild Ovarian Stimulation for IVF: An Alternative Strategy for Poor Prognosis Patients

**Published:** 2020

**Authors:** Ivan Sini, Arie A Polim, Nining Handayani, Adinda Pratiwi, Rosalina Thuffi, Nuraeni Yusup, Arief Boediono

**Affiliations:** 1- Morula IVF Jakarta Clinic, Jakarta, Indonesia; 2- IRSI Research and Training Center, Jakarta, Indonesia; 3- Department of Obstetrics and Gynecology, School of Medicine and Health Sciences, Atmajaya Catholic University of Indonesia, Jakarta, Indonesia; 4- Department of Anatomy, Physiology and Pharmacology, IPB University, Bogor, Indonesia

**Keywords:** Embryo freezing, In vitro fertilization, Mild ovarian stimulation, Poor ovarian response

## Abstract

**Background:**

Management of Poor Ovarian Reserve (POR) in in vitro fertilization remains a difficult challenge. The purpose of this retrospective cohort study was to compare the effectiveness of embryo banking strategy over a cohort of several mild stimulation cycles (Embryo Banking Strategy for Poor Prognosis/Embargo) to conventional full-dose antagonist protocol for IVF.

**Methods:**

Subjects identified as having poor ovarian response (POR) based on the Bologna criteria were recruited. In total, there were 113 subjects included in the analysis. Fifty-three subjects underwent embryo banking procedure (Embargo) protocol, and sixty subjects underwent the conventional full-dose antagonist protocol for IVF. The Chi-square test was used to compare the clinical pregnancy rate, miscarriage rate as well as live birth rate, while the Mann-Whitney U test was utilized to analyze the cost per clinical pregnancy between the two groups. A p<0.05 was considered statistically significant.

**Results:**

The two studied groups showed similar outcomes regarding clinical pregnancy rate, miscarriage rate, as well as live birth rate (p=0.966, p=0.310, and p= 0.469, respectively). Cost analysis of subjects who underwent mild ovarian stimulation followed by Embargo revealed the high cost of the protocol compared to conventional full-dose antagonist protocol ($10.507±6.181 *vs*. $9.533±2.530, p=0.002).

**Conclusion:**

The clinical outcomes of both protocols were comparable. Embargo procedure was not efficient in improving the overall clinical outcomes in patients who were expected poor ovarian responders as the protocol costed more comparing with conventional full-dose antagonist protocol. A larger prospective randomized control trial is needed to evaluate this finding.

## Introduction

The expected low probability of pregnancy in patients with Poor Ovarian Response (POR) has drawn the attention of experts to seek treatment options that might improve outcomes (1–4). POR is characterized by the low number of ovarian follicular pool and typically correlated with low oocytes quality (5). However, to date, the definition of POR has not yet been fully established due to ongoing debates regarding its classification and the expected outcomes.

Recent reports indicated a correlation between POR and low success rates of pregnancy following IVF treatment (6, 7). To achieve higher clinical outcomes in POR, several stimulation protocols have been introduced (3, 4). Currently, the most common approach is using conventional full-dose antagonist protocol (8, 9). This protocol allows us to increase the number of oocytes obtained with increasing doses of gonadotropins (10). On the other hand, practically, mild stimulation protocols were also introduced in patients who were expected poor responders. These protocols, which typically use lower doses of exogenous gonadotropins combined with oral agents such as clomiphene citrate (CC) and/or aromatase inhibitor, have risen in popularity and have become an alternative regimen with comparable clinical pregnancy rates (8, 11, 12).

According to the International Society for Mild Approaches in Assisted Reproduction (ISMAAR), mild stimulation is a regimen that aims for a minimum amount of 2–7 harvested oocytes per cycle. It has been understood that each menstrual cycle will have a different pool of antral follicles (13). Thus, it is possible to expect that each cycle will have a cohort variation of good quality oocytes. Alper and Fauser (2017) (14) predicted that comparable cumulative pregnancy rates in mild stimulation may be achieved after several cycles. Besides, given the nature of mild stimulation, folliculogenesis occurs with a minimum amount of FSH. Hence, the expected number of embryo per initiated cycle will also be low. Consequently, it is reasonable to consider collecting or accumulating the top-quality embryos by using embryo banking strategy over a cohort of several mild stimulation cycles (Embryo Banking Strategy for Poor Prognosis/Embargo) to indicate cycle-to-cycle variation.

Embargo protocol was designed to collect good quality embryos for elective frozen embryo transfer (e-FET) using mild stimulation protocol. As vitrification technology has now improved remarkably, the clinical decision to “freeze all” embryos may be implemented liberally in mild stimulation protocol (1). In controlled ovarian stimulation, the use of oral agent such as clomiphene citrate has been associated with impairment of endometrial receptivity. Therefore, embargo protocol might be a useful strategy to overcome the adverse effect of mild ovarian stimulation on the endometrial environment and receptivity.

To date, there have been no reports upon the use of mild stimulation protocol followed by Embargo protocol, to manage patients with POR. Therefore, this study was conducted and aimed to evaluate the clinical outcomes as well as cost analysis of the combined protocol compared to the conventional full-dose antagonist protocol.

## Methods

### Ethical approval, study population, and study design:

This single site, retrospective cohort study was approved by the local research ethics committee in the Faculty of Medicine, University of Indonesia, Jakarta (number: 92/UN2.F1/ETHIC/2019). Subjects underwent embargo protocol and conventional full-dose protocol during January 2016- till December 2018 period. A total of 113 women with expected poorer IVF outcomes were involved in the present study that consisted of fifty-three subjects who underwent embargo protocol and sixty subjects who went for the conventional full-dose antagonist protocol. In both groups, studied subjects were recruited using total sampling method based on our electronic medical database. The study was conducted at Morula IVF Jakarta among subjects identified as having poor ovarian response (POR) according to Bologna criteria (2011) with modified age criteria of ≥40 to ≥38 years old. Subjects had to meet at least 2 of the 3 Bologna criteria: (1) advanced maternal age (≥38 years), (2) previous history of poor ovarian response (≤3 oocytes with a conventional stimulation protocol), and (3) abnormal ovarian response test (antral follicle count <5–7 and or anti-Müllerian hormone <0.5–1.1 *ng/ml* [<3.6–7.9 *nmol/l*]). Exclusion criteria included the history of no oocyte retrieval, natural cycle protocol, existing endometriosis or ovarian cysts and endometrial pathology.

### Treatment protocol:

Embargo protocol consisted of several cycles of mild stimulation regimen. Subjects were given clomiphene citrate 150 *mg* from the 2nd day until the 6th day of menstrual cycle, followed by 150 *IU* HMG and antagonist injections from the 7th day, until leading follicles had reached a minimum diameter of 18 *mm*. Trigger injection was administered by giving 250 *mcg* rHCG and OPU was performed 36 *hr* later. Three hours after oocytes retrieval, insemination was conducted by ICSI or IMSI method. Subsequently, the resulting embryos were cultured up to day 3 (D3). Top-quality embryos were all vitrified on D3.

Embryo assessment at cleavage stage was performed manually under an inverted microscope, based on the Society for Assisted Reproductive Technology (SART) grading system (15). Embryo quality was divided into three categories of good, fair, and poor quality. The cell number or duplication (At least 7–8 cells on D3), fragmentation level (Less than 10%), as well as size and regularity of each blastomere were evaluated. The top-quality embryo was defined as good embryo that met three or two criteria.

Vitrification was started by immersing the embryo to equilibration medium containing 10% of ethylene glycol (Sigma). Once the equilibrium state was achieved, the embryo was immersed immediately into vitrification solution with composition of 15% ethylene glycol and 15% dimethyl sulfoxide (DMSO, Sigma). Hemi-straw was used as a carrier of embryo during storage in liquid nitrogen. Once there were two to three embryos, frozen embryo transfer (FET) was commenced, either by natural ovulation cycle monitoring or hormone replacement therapy. Embryo warming was performed by immediately immersing the hemi-straw containing the embryo into decreasing concentration of sucrose (0.5 *M*, 0.25 *M*, and 0.1 *M*). Assisted hatching was performed for occurrence of zona hardening due to vitrification.

Subjects underwent antagonist protocol were given rFSH/rLH 300 *IU*/150 *IU* from day 2 or 3 of menstrual cycle. Antagonist injections were commenced on day 5 of stimulation. Subsequently, trigger injection was administered by giving 250 *mcg* of rHCG. All embryos were cultured up to D3 and transferred on the same cycle. In accordance with standard operating procedures (SOP) regarding embryo transfer policy in our private clinic, a poorer quality embryo was not recommended for transfer. In both embargo and conventional full-dose antagonist protocols, luteal phase progesterone supplement was given in accordance with the Morula IVF clinic protocol.

### Outcomes:

The primary outcomes included the evaluation of clinical pregnancy rates, miscarriage, live birth rate, and cost analysis per started cycle resulting in clinical pregnancy. Clinical pregnancy is defined as a pregnancy confirmed by ultrasound scan or clinical documentation of at least one fetus with a discernible heartbeat (16). Miscarriage is defined as the condition in which pregnancy failed to develop in the first 20 weeks, including spontaneous abortion, incomplete abortion, and missed abortion. Cost analysis was calculated based on the clinical expenses, including the clinic administration fee, consultation fee, laboratory examination, stimulation drugs, ovum pick up operation up to embryo transfer, and luteal phase supplements until clinical pregnancy was detected. In embargo protocol, there was an additional cost for embryo banking and storage. The secondary outcomes included the evaluation of total amount of gonadotropin (IU), number of oocytes retrieved, number of mature oocytes, number of fertilized oocytes, number of embryos obtained on D3, number of top-quality embryos on D3, number of transferred embryos, and multiple pregnancies. In embargo protocol, secondary outcomes were calculated as cumulative value of mild stimulation up to three cycles.

### Data analysis:

Statistical analysis was performed using the Statistical Package for the Social Sciences (Release 20.0, SPSS, Inc.). Baseline characteristics of both groups were presented by percent-ages for categorical variables and by median and interquartile range for numerical variables due to non-normal distribution of data. Missing values within the clinical characteristic variables were managed by pair-wise method and were assured not to affect the primary or secondary outcomes. Chi-square test was used to analyze all categorical variables, whereas t-test or Mann-Whitney U test was used to analyze numerical variables. Significant results were indicated by a p-value of less than 0.05. Multivariate analysis was then performed to adjust potential confounders that may bias the clinical outcomes as the primary outcome. In embargo protocol, pregnancy rates were measured per frozen embryo transfer cycle when clinical pregnancy had been achieved.

## Results

### Patient characteristics:

Similar characteristics including age, BMI, basal FSH level, basal estradiol level, AMH, AFC, and infertility duration were found in 60 subjects who underwent conventional full-dose antagonist and 53 subjects who underwent embargo protocol as shown in table 1.

**Table 1.** Clinical characteristics of both groups

**Parameters**	**Embargo protocol (n=53)**	**Conventional full-dose antagonist (n=60)**	**p-value**
**Age (years)**	40±4.5	40±3.8	0.866
**BMI (*kg/m*^2^)**	23.10±5.59	23.73±6.08	0.146
**Basal FSH (*mIU/ml*)**	10.60±3.29	10.28±5.25	0.241
**Basal estradiol (*pg/ml*)**	38.05±30	37.87±22	0.393
**AMH (*ng/ml*)**	0.47±0.64	0.60±0.63	0.130
**AFC**	3±3	4±2	0.098
**Infertility duration (years)**	8±8	8.5±6.75	0.695

Note: Data were presented as median±IQR

### Primary outcomes:

Compared to embargo protocol, subjects who underwent conventional full-dose antagonist tend to achieve better clinical pregnancy rates although not statistically significant (26.67% *vs*. 22.64% respectively, RR of 0.986 and 95% CI (0.505–1.925), p=0.966). Similarly, miscarriage and live birth rate were comparable between groups with a trend of lower miscarriage rate and higher live birth rate in embargo group. Despite that, the cost analysis between the two groups shows a significant difference ($10.507± 6.181 *vs*. $9.533±2.530, respectively, p=0.002) (Table 2). Of all the subjects underwent embargo protocol, 66,03% (35/53) achieved their 2 or 3 top-quality embryos in the first mild stimulation cycle, 28.31% (15/53) in the second and 5,66% (3/53) in the third mild stimulation cycles.

**Table 2.** Primary outcomes between groups

**Outcome**	**Embargo protocol (n=53)**	**Conventional full-dose antagonist (n=60)**	**p-value**	**RR (95%CI)**
**Clinical pregnancy rates ^a^**	12 (22.64%)	16 (26.67%)	0.966	0.986 (0.505–1.925)
**Miscarriage ^a^**	1 (1.89%)	6 (10%)	0.310	0.290 (0.027–3.15)
**Live birth rate ^a^**	11 (20.75%)	10 (16.67%)	0.469	1.34 (0.606–2.962)
Cost analysis per clinical pregnancy achieved ^*^	$10.507±6.181	$9.533±2.530	0.002	-

Note:

a = data are presented as number of subjects and percentage (n (%)),

* = Cost analysis was calculated as cumulative cost from several (Median: 1±1) mild stimulations performed in USD

Multivariate analysis was performed to assess the potential confounder in clinical pregnancy rates, miscarriage rate, and live birth rate. The results showed that the risk to achieve clinical pregnancy, live birth, as well as miscarriage, did not differ between groups after adjusting for age parameter, basal FSH, estradiol, AMH levels, and AFC. In this study, none of these variables were linked to risk factors for clinical outcomes.

### Secondary outcomes:

As expected, subjects who underwent conventional full-dose antagonist protocol significantly received more gonadotropins during stimulation (Table 3). The median was 2700±600 *IU* per stimulation compared to the cumulative median of mild stimulation protocol (750±750 *IU*). Conventional full-dose antagonist protocol showed its superiority in the number of oocytes retrieved and number of matured oocytes following ICSI or IMSI method. However, the median of the total number of fertilized oocytes, embryos obtained on D3, top-quality embryos, and the number of embryos available for transfer were found comparable in both groups. In embargo group, 66.03% of subjects managed to obtain two to three top-quality embryos in the first mild stimulation cycle [Median (range)=1(1–3)]. In the conventional full-dose antagonist group, three patients (5%) had multiple pregnancies, whereas none of embargo groups had multiple pregnancies.

**Table 3.** Secondary outcomes between groups

**Parameters**	**Embargo protocol (n=53) ^c^**	**Conventional full-dose antagonist protocol (n=60)**	**p-value**	**RR**
**Total gonadotropins (IU) ^a^**	750±750	2700±600	<0.001	
**Retrieved oocytes ^a^**	3.00±2.00	3.00±4.00	0.029	
**MII oocytes following ICSI or IMSI method ^a^**	2.00±2.00	3.00±2.00	0.048	
**Fertilized oocytes ^a^**	2.00±2.00	2.00±2.00	0.670	
**Embryos obtained on D3 ^a^**	2.00±2.00	2.00±2.00	0.670	
**Top-quality embryos on D3 ^a^**	2.00±1.00	1.00±2.00	0.227	
**Embryo transferred/cycle ^a^**	2.00±1.00	1.00±1.00	0.478	
**Multiple pregnancy ^b^**	0 (0%)	3 (5%)	0.246	0.950 (0.896–1.007)

Note:

a = Data are presented as median ±IQR;

b = Data are presented as number of subjects and percentage (n (%));

c =Data are presented as cumulative value of several cycles (Median: 1±1)

## Discussion

In this retrospective study, our current findings become important in demonstrating the use of combined mild stimulation protocol with a “freeze-all” strategy—the so-called embargo protocol—to manage subjects with POR. Reed et al. (1) were the first authors who envisioned an alternative approach to consider the ‘freeze-all’ policy in subjects with POR. The basic concept of intercycle variation of oocyte quality may replace the concept of “once-off” treatment in conventional antagonist protocol. To our knowledge, this is the first study designed to answer this challenge.

Clinically, there are several issues regarding minimal stimulation IVF management in poor prognosis patients. The first problem is related to the low number of oocytes retrieved or lower quality of oocytes. These two parameters are most often reported during mild stimulation and even during conventional stimulation in subjects with POR (10, 17). Consequently, there may be very few numbers of top-quality embryos obtained (18). Based on this fact, embargo protocol allows subjects to generate more top-quality embryos for freezing.

Second, the anti-estrogenic effect due to the use of clomiphene citrate may impair endometrial thickness by compromising the endometrial proliferation (8, 10). Besides, there is a paradigm shift in the use of FET instead of fresh ET cycles. The use of FET cycles with controlled ovarian stimulation is growing due to less adverse effect on the endometrial lining, improved vitrification, and higher live birth rates compared to fresh ET (19). Thus, delaying embryo transfer for freezing and performing scheduled frozen embryo transfer in the natural cycle is a reliable alternative. Third, the disappointment and anxiety of subjects with POR who have experienced recurrent pregnancy failure after embryo transfer must be considered. Accordingly, embargo strategy could prevent high drop-out rates, defined as unreturned subjects following an embryo transfer failure.

Even though all the advantages of embargo protocol are mentioned above, our current study could not prove the superiority of embargo protocol over conventional full-dose antagonist. In this study, overall pregnancy outcomes, including clinical pregnancy, miscarriage as well as live birth rate were found similar in studied groups. Clinical pregnancy rates in embargo protocol were found comparable to the conventional full-dose antagonist protocol. Revelli et al. (8) reported similar clinical pregnancy rates between single mild stimulation and “long-protocol” of GnRH agonist followed by fresh transfer (23.2% *vs*. 19.9%, respectively) whereas our study showed comparable clinical pregnancy rates from cumulative mild stimulation (Table 2, median 1±1). However, the trend in lower miscarriage rate was found in embargo group. Through a prospective randomized study, abortion rate in single mild stimulation group followed by fresh transfer was approximately 23.4% and 40%, respectively (8, 10). It is assumed that our result showed the potential benefit of intercycle variation concept that may recruit better quality oocytes for reducing the miscarriage rate. Larger prospective randomized control trial studies are needed to confirm this finding.

With respect to the live birth rate, as a critical parameter of reproductive outcome, no significant difference was found in both groups. Nevertheless, the trend of higher live birth rate in embargo protocol is warranted to reevaluate our approach in IVF stimulation by relying on one cycle of treatment. Supporting the result of our embargo approach, accumulation of oocytes from several cycles in patients with poorer ovarian response resulted in the live birth rate of approximately 30.2% (2). Regarding the similar concept of embryo pooling, our result shows findings contrary to that of Çelik et al.; when compared to fresh embryo transfer, the live birth rate in the pooling method was significantly lower (25% *vs*. 14%, respectively, p=0.04) (20). The authors, however, did not elucidate the reason behind the clinical decision for embryo pooling, whereas freeze-all decision in embargo group was implemented to avoid the negative effect of clomiphene citrate which expected to impair the endometrial receptivity.

Although conventional full-dose antagonist group consumed more gonadotropin than embargo protocol group, practical cost to achieve clinical pregnancy was higher in embargo protocol group. Repeated ovum pick up procedure was the main contributor to higher cost in this group. In terms of cost analysis, our current finding supported the result that embryo pooling or banking had led to increased cost of treatment (20).

Due to the nature of this retrospective design, the study had several limitations. Several potential confounders were present and it was impossible to control them all. Also regarding cost analysis, only direct costs for IVF program were calculated. The indirect costs such as accommodation, psychology consultation, acupuncture, or other costs that were related to infertility treatment were not calculated. For this reason, the terminology of cost analysis was used instead of cost-effectiveness. In addition, cost analysis per live birth could not be provided due to lack of follow up data concerning the cost since only half of the studied subjects continued to have checkup in our clinic after clinical pregnancy was detected.

## Conclusion

Both protocols were comparable in overall pregnancy outcomes. Despite its milder approach, embargo protocol was shown to cost more when compared to full-dose antagonist. Larger prospective RCT is needed to evaluate the efficiency of this alternative option for poor prognosis patients.

## References

[1] Reed BG Babayev SN Bukulmez O Shifting paradigms in diminished ovarian reserve and advanced reproductive age in assisted reproduction: Customization instead of conformity. Semin Reprod Med. 2015;33(3):169–78. 26036898 10.1055/s-0035-1552585

[2] Cobo A Garrido N Crespo J José R Pellicer A Accumulation of oocytes: a new strategy for managing low-responder patients. Reprod Biomed Online. 2012;24(4):424–32. 22386762 10.1016/j.rbmo.2011.12.012

[3] Ubaldi FM Rienzi L Ferrero S Baroni E Sapienza F Cobellis L Management of poor responders in IVF. Reprod Biomed Online. 2005;10(2):235–46. 15823231 10.1016/s1472-6483(10)60946-7

[4] Loutradis D Drakakis P Vomvolaki E Antsaklis A Different ovarian stimulation protocols for women with diminished ovarian reserve. J Assist Reprod Genet. 2007;24(12):597–611. 18034299 10.1007/s10815-007-9181-2 PMC3455002

[5] Jirge PR Poor ovarian reserve. J Hum Reprod Sci. 2016;9(2):63–9. 27382229 10.4103/0974-1208.183514 PMC4915288

[6] Broer SL van Disseldorp J Broeze KA Dolleman M Opmeer BC Bossuyt P Added value of ovarian reserve testing on patient characteristics in the prediction of ovarian response and ongoing pregnancy: an individual patient data approach. Hum Reprod Update. 2013;19(1):26–36. 23188168 10.1093/humupd/dms041

[7] van Loendersloot LL van Wely M Limpens J Bossuyt PM Repping S van der Veen F Predictive factors in in vitro fertilization (IVF): a systematic review and meta-analysis. Hum Reprod Update. 2010;16(6):577–89. 20581128 10.1093/humupd/dmq015

[8] Revelli A Chiadò A Dalmasso P Stabile V Evangelista F Basso G “Mild” vs. “long” protocol for controlled ovarian hyperstimulation in patients with expected poor ovarian responsiveness undergoing in vitro fertilization (IVF): a large prospective randomized trial. J Assist Reprod Genet. 2014;31 (7):809–15. 24700398 10.1007/s10815-014-0227-y PMC4096882

[9] Tarlatzis BC Zepiridis L Grimbizis G Bontis J Clinical management of low ovarian response to stimulation for IVF: a systematic review. Hum Reprod Update. 2003;9(1):61–76. 12638782 10.1093/humupd/dmg007

[10] Siristatidis C Salamalekis G Dafopoulos K Basios G Vogiatzi P Papantoniou N Mild versus conventional ovarian stimulation for poor responders undergoing IVF/ICSI. In Vivo. 2017;31(2):231–7. 28358705 10.21873/invivo.11050 PMC5411750

[11] Bastu E Buyru F Ozsurmeli M Demiral I Dogan M Yeh J A randomized, single-blind, prospective trial comparing three different gonadotropin doses with or without addition of letrozole during ovulation stimulation in patients with poor ovarian response. Eur J Obstet Gynecol Reprod Biol. 2016; 203:30–4. 27236602 10.1016/j.ejogrb.2016.05.027

[12] Goswami SK Das T Chattopadhyay R Sawhney V Kumar J Chaudhury K A randomized single-blind controlled trial of letrozole as a low-cost IVF protocol in women with poor ovarian response: a preliminary report. Hum Reprod. 2004;19 (9):2031–5. 15217999 10.1093/humrep/deh359

[13] Deb S Campbell BK Clewes JS Pincott-Allen C Raine-Fenning NJ Intracycle variation in number of antral follicles stratified by size and in endocrine markers of ovarian reserve in women with normal ovulatory menstrual cycles. Ultrasound Obstet Gynecol. 2013;41(2):216–22. 22744915 10.1002/uog.11226

[14] Alper MM Fauser BC Ovarian stimulation protocols for IVF: is more better than less? Reprod Biomed Online. 2017;34(4):345–53. 28169189 10.1016/j.rbmo.2017.01.010

[15] Racowsky C Stern JE Gibbons WE Behr B Pomeroy KO Biggers JD National collection of embryo morphology data into society for assisted reproductive technology clinic outcomes reporting system: associations among day 3 cell number, fragmentation and blastomere asymmetry, and live birth rate. Fertil Steril. 2011;95(6):1985–9. 21411078 10.1016/j.fertnstert.2011.02.009

[16] Zegers-Hochschild F Adamson GD Dyer S Racowsky C de Mouzon J Sokol R The international glossary on infertility and fertility care, 2017. Hum Reprod. 2017;32(9):1786–801. 29117321 10.1093/humrep/dex234 PMC5850297

[17] Karande V Gleicher N A rational approach to the management of low responders in in-vitro fertilization. Hum Reprod. 1999;14(7):1744–8. 10402380 10.1093/humrep/14.7.1744

[18] Shrestha D La X Feng HL Comparison of different stimulation protocols used in in vitro fertilization: a review. Ann Transl Med. 2015;3(10):137. 26207230 10.3978/j.issn.2305-5839.2015.04.09 PMC4486909

[19] Shapiro BS Daneshmand ST Garner FC Aguirre M Hudson C Clinical rationale for cryopreservation of entire embryo cohorts in lieu of fresh transfer. Fertil Steril. 2014;102(1):3–9. 24842675 10.1016/j.fertnstert.2014.04.018

[20] Çelik S Turgut NE Cengiz Çelik D Boynukalın K Abal R Purisa S The effect of the pooling method on the live birth rate in poor ovarian responders according to the Bologna criteria. Turk J Obstet Gynecol. 2018;15(1):39–45. 29662715 10.4274/tjod.62447 PMC5894535

